# Elevated Circulatory Levels of Microparticles Are Associated to Lung Fibrosis and Vasculopathy During Systemic Sclerosis

**DOI:** 10.3389/fimmu.2020.532177

**Published:** 2020-10-23

**Authors:** Damien Leleu, Emeline Levionnois, Paoline Laurent, Estibaliz Lazaro, Christophe Richez, Pierre Duffau, Patrick Blanco, Vanja Sisirak, Cecile Contin-Bordes, Marie-Elise Truchetet

**Affiliations:** ^1^ University of Bordeaux, CNRS, ImmunoConcEpT, UMR 5164, Bordeaux, France; ^2^ Immunology and Immunogenetic Department, Bordeaux University Hospital, Bordeaux, France; ^3^ Internal Medicine Department, Bordeaux University Hospital, Bordeaux, France; ^4^ Centre national de reference des maladies auto-immunes systémiques rares de l’Est et du Sud-Ouest (RESO), Bordeaux, France; ^5^ Rheumatology Department, Bordeaux University Hospital, Bordeaux, France

**Keywords:** microparticles (MPs), systemic sclerosis (scleroderma), fibrosis, platelets, endothelial cells (ECs), immunosuppressive agent

## Abstract

**Background:**

Microparticles (MPs) are vesicular structures that derive from multiple cellular sources. MPs play important roles in intercellular communication, regulation of cell signaling or initiation of enzymatic processes. While MPs were characterized in Systemic Sclerosis (SSc) patients, their contribution to SSc pathogenesis remains unknown. Our aim was to investigate the potential role of MPs in SSc pathophysiology and their impact on tissue fibrosis.

**Methods:**

Ninety-six SSc patients and 37 sex-matched healthy donors (HD) were enrolled in this study in order to quantify and phenotype their plasmatic MPs by flow cytometry. The ability of MPs purified from SSc patients and HD controls to modulate fibroblast’s extra-cellular matrix genes expression was evaluated *in vitro* by reverse transcriptase quantitative polymerase chain reaction.

**Results:**

SSc patients exhibited a higher concentration of circulatory MPs compared to HD. This difference was exacerbated when we only considered patients that were not treated with methotrexate or targeted disease-modifying antirheumatic drugs. Total circulatory MPs were associated to interstitial lung disease, lung fibrosis and diminished lung functional capacity, but also to vascular involvement such as active digital ulcers. Finally, contrary to HD MPs, MPs from SSc patients stimulated the production of extracellular matrix by fibroblast, demonstrating their profibrotic potential.

**Conclusions:**

In this study, we provide evidence for a direct profibrotic role of MPs from SSc patients, underpinned by strong clinical associations in a large cohort of patients.

## Background

Systemic Sclerosis (SSc) is a rare and complex autoimmune disease that involves immune activation, microvascular dysfunction and perivascular fibrosis affecting both the skin and internal organs. SSc is associated with serious complications that severely impact patients’ quality of life and vital prognosis. Among them are vasculopathies (Raynaud’s phenomenon, digital ulcers), gastrointestinal complications, cardiovascular damage (pulmonary arterial hypertension, cardiac fibrosis) and interstitial lung disease (ILD) that can lead to pulmonary fibrosis. The pathophysiology of SSc remains poorly understood, however the systemic nature of the disease may be due, in part, to aberrant immune stimulation, as well as to platelets and endothelial cells hyperactivation (1, 2). Microparticles (MPs) generated from these activated immune cells, platelets and endothelial cell, were shown in inflammatory and autoimmune diseases to display pathogenic properties across multiple tissues, and may as well contribute to SSc pathogenesis (3).

Virtually all eukaryotic cells shed submicron vesicles constitutively, upon activation or during apoptosis. It is only recently that their potential roles in physiology and pathophysiology have been appreciated, including their involvement in blood coagulation, inflammation, and intercellular signaling (3). MPs are vesicular structures that derive from multiple cellular sources and in the circulation, they notably originate from platelets (4, 5) and endothelial cell (6). There are two principal types of MPs identified according to their size and content. Large MPs (0.1 to 1 µm) that arise from outward budding of the plasma membrane, and small MPs also called exosomes (50 to 150 nm) which are manufactured within multivesicular bodies of the endocytic tract (7). MPs can play many roles in intercellular communication, regulation of cell signaling or initiation of enzymatic processes (3). Interestingly, large extracellular MPs were shown as a major carrier of self-DNA and thus may represent an important self-antigen involved in the loss of tolerance mechanisms, and the development of autoimmune diseases, when improperly cleared (8). Therefore, MPs represent functional units with a disseminated storage pool of bioactive effectors that are starting to be recognized as important players in different autoimmune diseases including Systemic Lupus Erythematosus (SLE) and SSc (9–11). MPs can originate from every type of cells, particularly under stress conditions. Endothelial cell (EC) damage is one major hallmark of SSc and lead to the shedding of endothelial MPs [EMPs; (12, 13)]. *Batteux* et al. showed in a murine model of SSc, that inhibiting MPs shedding either chemically or genetically diminishes the hallmarks of oxidative and endothelial stress but also skin and lung fibrosis (14). However, the underlying mechanisms of MPs impact on SSc pathogenesis in these animal models were not characterized. Nomura et al. measured circulatory MPs originating from platelets (PMPs) and monocytes, on a small cohort of SSc patients with pulmonary disease, and showed that they were both increased compared to healthy donors [HD; (15)]. Furthermore, Maugeri et al. have recently shown that PMPs that accumulate in SSc patients promote neutrophil autophagy and the formation of neutrophil extracellular traps (NETs) upon stimulation by PMPs-associated HMGB1 [High-Mobility Group Box 1; (16)]. However, associations between PMPs, their content in HMGB1, circulatory NETs byproducts and specific SSc clinical features were not described. It is only recently that pulmonary arterial hypertension (PAH) in a very small cohort of SSc patients was associated with elevated levels of circulatory EMPs. Such contribution of EMP to PAH was proposed to rely on SSc patients MPs ability to stimulate EC capacity to produce inflammatory cytokines (17). Furthermore, MPs containing mitochondrial DNA were recently suggested to contribute to ILD in SSc patients through their ability to activate inflammatory immune responses (18). Although these studies support the importance of MPs in SSc pathophysiology, human data on MPs in SSc and their involvement in fibrotic process is remain scarce.

Taking into account the previously described involvement of platelets and endothelial cell in the SSc physiopathology (1, 2) and the previous observation of the total MPs, EMPs and PMPs elevations during SSc (10, 16, 17), we decided to analyze all circulatory MPs and those derived from platelet and endothelial cells, in our patient cohort. Our objective was to investigate total numbers of these MPs in SSc patients compared to HD, and to study whether MPs counts were associated to specific clinical features. We also investigated the impact of purified total MPs from SSc patients and HD on fibroblasts production of extracellular matrix components. We report evidence for a direct profibrotic role specifically of SSc patients’ MPs *in vitro* underpinned by clear clinical associations between SSc patients’ MPs numbers with lung involvement in a large cohort.

## Methods

### Study Population

Patients with SSc have been included in the biomedical research project “MICROLUPS” (Microparticles in lupus and SSc), approved by the institutional ethics committee (CPP, SUD-EST IV, 18/024) at the university hospital of Bordeaux. All patients satisfied the American College of Rheumatology/European League Against Rheumatism 2013 classification criteria for SSc (19) and provided written informed consent before inclusion. Clinical (age, sex, disease duration, skin disease, articular disease, heart disease, lung disease, kidney disease, and gastrointestinal disease) and biologic (anti-nuclear serology, creatinine clearance) features of each patient were recorded, as well as their respiratory function tests and treatments. Values of the modified Rodnan skin score (MRSS) and right ventricular systolic pressure (RVSP), that were recorded were the one that were the closest to the collection time of the sample. ILD was diagnosed if respiratory function tests showed a restrictive effect with a decrease in carbon monoxide diffusion capacity (DLCO < 80% and/or FVC < 70%) associated with damages (such as ground-glass opacities, honeycombing or reticular infiltrations) on high-resolution computed tomography (HRCT) and stratified into limited and extensive ILD, according to Goh et al. and Roofeh D. et al. (20, 21). Diagnosis of PAH was based on the assessment of mean pulmonary arterial pressure (PAPm > 25mmHg) by right heart catheterization (22). Severe SSc phenotypes were defined as SSc with ILD, PAH, cardiac disease, renal crisis, and active digital ulcers. The control group consisted of sex‐matched healthy donors (mean age = 45 years; 72,72% of women) obtained from the local Blood Transfusion Center (Etablissement Français du Sang, University Hospital of Bordeaux).

### Pre-Analytical Sample Processing

SSc Patients and HD’s blood were collected in 7 ml EDTA tubes, without applying the tourniquet and with a needle of at least 21 gauges. Samples were kept upright and rapidly conveyed to the laboratory without being shaken and then processed within 2 h of collection. Blood was gently transferred into a 15 ml tube and spun at 3500g for 15 minutes (without brakes). Platelet-poor plasma (PPP) was gently collected leaving a minimum of 1 ml of plasma above the pellet. The PPP was then spun again at 3500g for 15 minutes (without brakes). Platelet-free plasma (PFP) was collected leaving a minimum of 500 µl above the platelet pellet. PFP was then aliquoted in 1 ml aliquots and stored at −80°C.

### Microparticles Phenotyping

Patient and control samples of PFP were thawed at room temperature and spun at 21,000 g for 1 h at 4°C. The pellet containing MPs was resuspended in 1 ml of 0.1µm filtered Phosphate Salt Buffer (PBS) and 100 µl of this suspension was labeled with 5 ng/µl of anti-CD235a Pacific Blue (Mouse IgG1, κ, Biolegend), anti-CD41 APC (Mouse IgG1, κ, Biolegend), anti-CD31 PE-Cy7 antibodies (Mouse IgG1, κ, Biolegend), and lactadherin (Hematologic Technologies, diluted at 1/50) or with 5 ng/µl anti-CD45 FITC (Mouse IgG1, Beckman Coulter), anti-CD66b PerCP (Mouse IgG1, κ, Biolegend), anti-CD3 BV510 (Mouse IgG1, κ BD biosciences) or with anti-CD19 PE (Mouse IgG1, Beckman Coulter) for 30 minutes, in the dark at room temperature. MPs count-beads^®^ (Biocytex, diluted at 1/10), for MPs counting, and 100 µl of filtered PBS were added after the labeling. Samples were then analyzed by flow cytometry on a FACS canto II (Becton Dickinson). Megamix-Plus SSC^®^ (Biocytex) was used to set up the threshold in side scatter (SSC) and place the MPs gate in accordance with the manufacturer’s protocol. A second threshold was set on forward scatter (FSC) using a tube containing only 0.1µm filtered PBS together with the antibody mix, according to the protocol from Burbano et al. (23). All the background noise generated by the buffer was thus eliminated. These settings allowed us to define the total MPs among which two sub-populations were characterized: the EMPs (CD235a^-^CD41^-^CD31^+^) and the PMPs (CD235a^-^CD41^+^).

### Fibroblast Culture With Microparticles

Fibroblasts extracted from mammoplasty were cultured in 24-well plates with 50,000 cells per well. Fibroblasts were allowed to adhere for 24 h in Dulbecco’s Modified Eagle Medium (DMEM, Gibco), containing 10% fetal bovine serum (FBS, GE Healthcare Bio-Sciences), 1% penicillin/streptomycin (Gibco) and 1% Pyruvate (complete DMEM medium). Then, the medium was replaced by the same medium without FBS overnight to starve the cells, in order to promote their stimulation.

Patient and control samples were thawed at room temperature and centrifuged at 21,000g for 1 h at 4°C to pellet the MPs. The supernatant was removed to leave only 50 µl above the MPs pellet. The MPs were then resuspended in 950 µl of filtered PBS and centrifuged again at 21,000g for 1 h at 4°C. The MPs concentration was adjusted after quantification by flow cytometry on the FACS Canto II (Becton Dickinson) with complete DMEM medium to obtain a concentration of 1,000 MPs/µl (corresponding to 10 times the cell concentration). MPs were then cultured with the fibroblasts at 10 MPs/µl, 100 MPs/µl, and 1,000 MPs/µl. As a positive control, fibroblasts were treated with 10 ng/ml of TGF-β (Miltenyi Biotec), which is known to induce collagen expression (**Figure S3**). Fibroblasts and MPs were co-cultured for 24 h before RNA extraction.

### Reverse Transcription Quantitative PCR (RT qPCR)

mRNA was purified from fibroblasts using an RNeasy Plus Micro Kit (Qiagen), and mRNA concentration and purity were quantified with a Spectrophotometer DS11 (Denovix). RNA integrity number (RIN) was assessed using an Agilent 2200 TapeStation (Agilent Technologies). All procedures were performed according to the manufacturer’s instructions. Total mRNA was converted to cDNA using GoScript Reverse Transcription (Promega TM). qPCR was performed using GoTaq Master Mix (Promega TM). The following targets were analyzed: 18s, RPLP0, Col1A1, Col1A2, MMP1and CCL2. The 18s-specific primers used were 5’-TGCCATCACTGCCATTAAG-3’ (forward) and 5’-TGCTTTCCTCAACACCACATG-3’ (reverse), the RPLP0-specific primers used were 5’-GCAGCATCTACAACCCTGAAG-3’ (forward) and 5’-CACTGGCAACATTGCGGAC-3’ (reverse), the Col1A1-specific primers used were 5’-CCCTCCTGACGCACGG-3’ (forward) and 5’-GTGATTGGTGGGATGTCTTCGT-3’ (reverse), the Col1A2-specific primers used were 5’-CTGTAAGAAAGGGCCCAGCC-3’ (forward) and 5’-GACCCCTTTCTCCACGTGG-3’ (reverse), the MMP1-specific primers used were 5’-GGAGGAAAAGCAGCTCAAGAAC-3’ (forward) and 5’-TCCAGGGTGACACCAGTGACT-3’ (reverse) and the CCL2-specific primers used were 5’-AACCACAGTTCTACCCCTGGG-3’ (forward) and 5’-TAATGATTCTTGCAAAGACCCTCAA-3’ (reverse). Samples were distributed in duplicate in a 384-well plate using an Epmotion 5073 automated pipetting system (Eppendorf). Real-time quantitative PCR was performed using a CFX384 thermocycler (Bio-Rad TM), the data were analyzed using Bio-Rad TM CFX Manager software (Bio-Rad TM). mRNA differential expression was evaluated according to the normalization of the mean housekeeping genes expression (18s and RPLP0) and the DMEM 10% FBS condition (ΔΔCt method: 2^-ΔΔCt^, ΔΔCt=Ct_target_ - Ct_mean 18s & RPLP0_ – Ct_target DMEM 10% FBS condition_).

### Statistical Analysis

Statistical analyses were performed using GraphPad Prism (La Jolla, CA). For populations who satisfied the Kolmogorov–Smirnov normality test, a two-tailed Student’s t-test for unpaired or paired samples and one-way repeated-measures ANOVA test followed by the Bonferroni correction were used to compare the different populations according to the experimental design. When the normality test was not satisfied, the Mann-Whitney, Wilcoxon and Kruskal Wallis tests were used. Correlations were analyzed using the Spearman test. A p-value <0.05 was considered statistically significant.

## Results

### Cohort Description

From December 2018 to December 2019, 96 SSc patients were included in this study. Approximately 2/3 of the cohort presented a limited form of the disease (n=57, 59%) while the rest of the patients had a diffuse form of the disease (n=39, 41%). The demographic characteristics and main clinical or biological features of the entire cohort are presented in **Table 1**. Thirty-two patients were treated with methotrexate, 22 patients received steroids, 13 patients received a targeted disease-modifying antirheumatic drugs (tDMARDs; eight Tocilizumab, three Rituximab, and two Baricitinib), 5 received mycophenolate mofetil (MMF), and 8 received Hydroxychloroquine (HCQ) at the time of collection. Thirty-seven sex-matched healthy donors (HDs) served as controls. Overall, healthy donors were on average younger than SSc patients (mean HD = 45 years *vs* mean SSc = 61 years). Nevertheless, MPs levels were previously reported as unaffected by age (24, 25) and when we analyzed total MPs, EMPs and PMPs in our cohorts of SSc patients and HD controls we haven’t observed any correlation between MPs counts and age (**Figure S1**). Therefore, we have a large cohort of SSc patients with multiples phenotypes and treatments that can be extensively analyzed and compared to HD controls.

**Table 1:** Main characteristics of SSc patients included in the study.

	lcSSc (n=57)	dcSSc (n=39)	All SSc patients (n=96)	P
**Age at onset, median ± SD years**	61 (13.68)	62 (10.88)	59 (12.87)	ns
**Gender (Female) (%)**	51 (80.95)	16 (41.03)	67 (73.63)	0.02
**Disease duration, mean ± SD years**	7.89 ± 7.34	7.5 ± 5.55	7.77 ± 6.53	ns
**MRSS, mean ± SD**	4 ± 5.11	16.93 ± 10.03	8.4 ± 8.01	<0.0001
**Active smokers (%)**	17 (26.98)	8 (20.51)	25 (27.47)	ns
**Active disease (%)**	8 (12.7)	8 (20.51)	16 (17.58)	0.0795
**Digestive disease (%)**	28 (44.44)	17 (43.59)	45 (49.45)	ns
**Cardiac involvement (%)**	4 (6.35)	2 (5.13)	6 (6.59)	ns
**Raynaud phenomenon (%)**	55 (93.65)	25 (64.10)	84 (92.31)	ns
**Digital ulcers (%)**	6 (9.52)	8 (20.51)	14 (15.38)	0.0286
**Synovitis/tenosynovitis (%)**	15 (23.81)	4 (41.02)	19 (20.88)	ns
**Interstitial lung disease (%)**	17 (26.98)	16 (41.02)	33 (36.26)	0.009
**TLC, mean ± SD**	1.01 ± 0.15	0.95 ± 0.18	0.98 ± 0.18	ns
**DLCO/VA, mean ± SD**	0.67 ± 0.16	0.61 ± 0.19	0.65 ± 0.18	ns
**FVC, mean ± SD**	1.13 ± 0.21	0.88 ± 0.24	1.05 ± 0.25	<0.0001
**PAPS, mean ± SD**	32.8 ± 9.05	33 ± 10.4	32.87 ± 10.92	ns
**ANA (%)**	57 (100%)	39 (100%)	96 (100%)	ns
**Anti-scl70 Ab (%)**	5 (7.94)	19 (48.71)	20 (21.98)	<0.0001
**Anti-centromeres Ab (%)**	39 (61.9)	2 (5.13)	41 (45.05)	<0.0001
**Anti-ARNpolIII Ab (%)**	1 (1.59)	3 (7.69)	4 (4.4)	0.0849
**Methotrexate (%)**	19 (33.33)	13 (33.33)	32 (33.33)	ns
**tDMARDs (%)**	6 (10.53)	7 (17.95)	13 (13.54)	ns

SSc, Systemic Sclerosis; lcSSc, limited cutaneous systemic sclerosis; dcSSc, diffuse cutaneous systemic sclerosis; SD, standard deviation; mRSS, modified Rodnan skin score; TLC, total lung capacity; DLCO, volume-corrected carbon monoxide diffusing capacity; FVC, forced vital capacity; PAPs, systolic pulmonary arterial pressure; ANA, Anti-Nuclear antibody; Ab, antibody; tDMARDs, targeted disease-modifying antirheumatic drugs; ns, not significant.

### Circulatory MPs Are Increased in SSc Patients, Especially Those of Originating From Platelets

**Figure 1:**
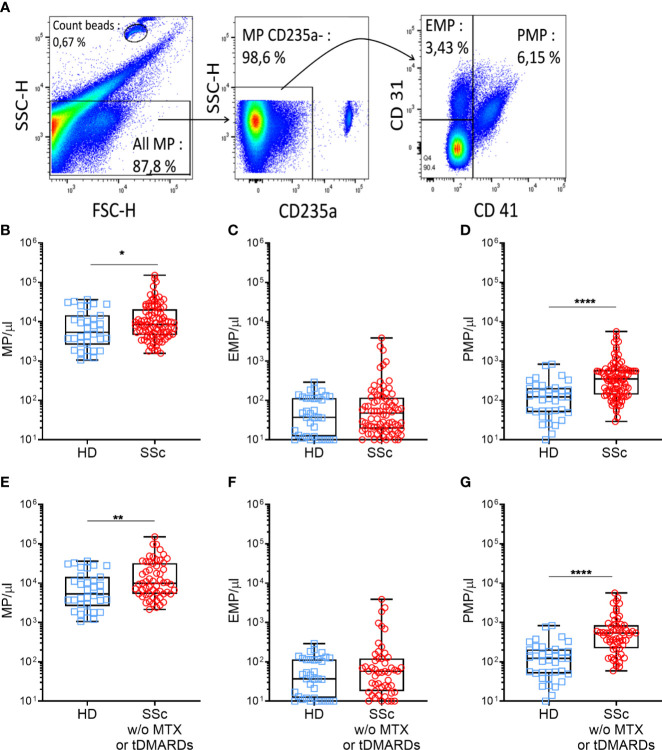
Circulating MPs are increased in SSc patients compared to HD. MPs were gated on their scatter property, then among the CD235a-, the PMPs (CD41+) and the EMPs (CD41-CD31+) were defined **(A)**. Plasma MPs **(B)**, EMPs **(C)**, PMPs **(D)**, levels were compared between HDs and SSc patients (HDs = 37, SSc = 96). Same comparison of MPs **(E)**, EMPs **(F)** and PMPs **(G)** levels between HDs and SSc was made, taking into account only those patients not treated with methotrexate or tDMARDs (HDs = 37, SSc = 60); Box-plots represent the extreme values, the first and third quartiles and the medians. *p < 0.05 **(B)**, **p < 0.01 **(E)**, ****p < 0.0001 **(D, G)** by Mann-Whitney. MPs, microparticles; EMPs, endothelial cells derived microparticles; PMPs, platelets derived microparticles; HD, healthy donors; SSc, systemic sclerosis; MTX, methotrexate; tDMARDS, targeted disease-modifying antirheumatic drugs.

Plasma levels of MPs were assessed by flow cytometry. Anti-CD45, CD3, CD19, and CD66b antibodies labeled only a very minor MP fraction and were thus not retained in subsequent experiments (data not shown). Representative gating strategy of blood MPs was shown in **Figure 1A**. Even though mean levels of total MPs were not significantly different between SSc patients and HDs (mean ± SEM of 16859 ± 2528 MPs/μl *vs.* 10408 ± 1827 MPs/μl, respectively, data not shown), a higher proportion of SSc patients exhibited increased levels of MPs compared to HD (median of 8444 *vs*. 5331, **Figure 1B**, p=0.04). More than half of the patients presented over than 8,000 MPs/μl vs. one third of HDs. This increase was independent of the mechanism leading to their generation, since both MPs originating from dying (phosphatidylserine (PS)^+^) (26) and living (PS^-^) cells were significantly increased in SSc patients compared to HD (median of 429.0 MPs/μl *vs*. 208.5 MPs/μl, and of 5042 MPs/μl *vs*. 2217 MPs/μl, respectively, p<0.05, data not shown). Among total MPs, we particularly analyzed those originating from platelets (PMPs) and endothelial cells (EMPs). These two populations were defined by the absence of red blood cell marker (CD235) expression. PMPs were further characterized by the expression of CD41, with or without CD31, and EMPs by the expression of CD31 without CD41 (**Figure 1A**). Although no overall difference in EMPs concentration between HD and SSc was observed (**Figure 1C**), PMPs concentration was significantly higher in SSc patients compared to HD (median of 352.0 *vs* 122.0 PMPs/μl, respectively, **Figure 1D**, *p*<0.0001). The numbers of these PMP were elevated in SSc patients regardless of their PS expression (median of 108 PMPs PS^-^/μl *vs*. 50 PMPs PS^-^/μl, and of 192 PMPs PS^+^/μl *vs*. 88 PMPs PS^+^/μl, respectively, p<0.001, data not shown).

We next investigated whether specific therapeutic regimens administered to SSc patients could impact MPs circulatory levels. As shown in **Figure S2A**, total MPs concentration did not differ between patients without immunosuppressive (IS) therapy (10512 MPs/µl), with corticosteroids (9379 MPs/µl), with non-specific immunosuppressive drugs including MMF (7188 MPs/µl), HCQ (9385 MPs/µl) or methotrexate (7693 MPs/µl) and those with tDMARDs (8878 MPs/µl). While total MPs (**Figure S2A**) and EMPs concentration (data not shown) were similar in treated and untreated patients, PMPs concentration was significantly decreased in patients treated with methotrexate and tDMARDs, compared to untreated patients (median of 230 PMPs/µl, 187 PMPs/µl, and 544 PMPs/µl, respectively, p<0.01, **Figure S2B**). Based on these observations and to avoid any confounding effect of the patient’s treatment regimens, we excluded from our initial comparison patients treated by methotrexate or tDMARDs (n=36). As shown in **Figures 1E, G**, we observed a greater increase in total MPs (mean ± SEM of 21567 ± 3686 MPs/μl *vs.* 10408 ± 1827 MPs/μl, respectively, p<0.01, **Figure 1E**) and PMPs (mean ± SEM of 828.6 ± 141.4 *vs* 167.0 ± 30.76 PMPs/μl, respectively, *p*<0.0001, **Figure 1G**) concentrations in SSc patients compared to HD controls. However, irrelevant of the treatment, EMPs concentration between SSc patients and HD controls remained comparable (**Figure 1F**). Altogether, these results indicate an increase of circulatory MPs in SSc patients, in particular of those with a platelet origin, questioning their association to specific clinical features of SSc patients and their functional role in SSc pathogenesis.

### High MPs Concentrations Are Associated With Severe SSc Phenotypes

**Figure 2:**
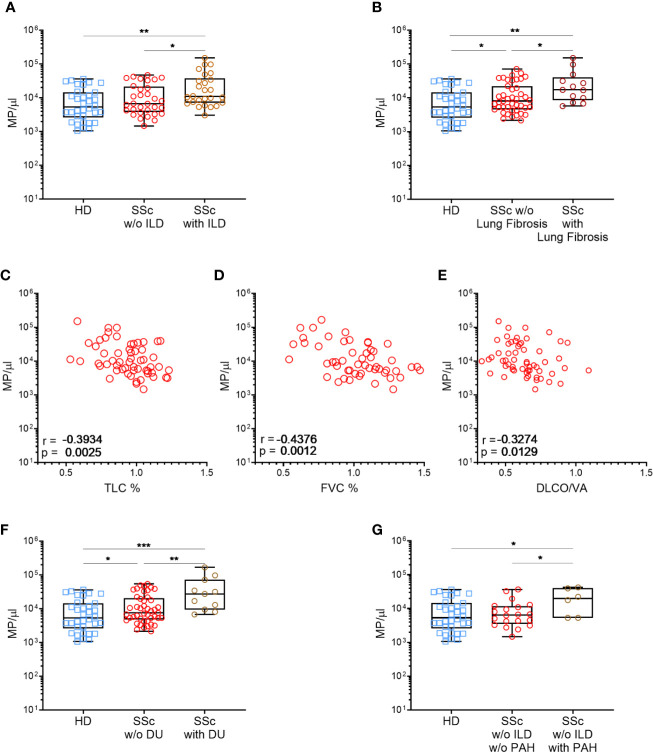
High MPs concentrations are associated with severe SSc phenotypes. Circulating MPs were compared between HD, patients with and without diffuse interstitial lung disease [Normal = 32, interstitial lung disease = 26; **(A)**], and lung fibrosis [HD = 37, SSc =43, SSc with lung fibrosis=13; **(B)**]. The concentration of circulating MPs is compared to the lung function test of SSc patients (SSc=59): TLC **(C)**, FVC **(D)** and DLCO/VA **(E)**. Circulating MPs were compared between HD, patients with or without vascular injury like digital ulcers [HD = 37, SSc =47, SSc with digital ulcers = 11; **(F)**] or pulmonary arterial hypertension without interstitial lung disease [HD = 37, SSc = 20, SSc with PAH = 6; **(H)**]. In **(A**, **B**, **F**, **G)** symbols represent individual subjects; Box-plots represent the extreme values, the first and third quartiles and the medians. In **(C–E)**, Correlations were determined using Spearman’s test. *p<0.05 by Mann-Whitney **(A–C, F–H)**, **p<0.01 by Mann-Whitney **(A–E, G)**, ***p<0,001 **(G)**. MPs, microparticles; HD, healthy donors; SSc, systemic sclerosis; ILD, interstitial lung disease; TLC, total lung capacity; FVC, forced vital capacity; DLCO/VA, diffusing capacity of the lung for carbon monoxide; DU, active digital ulcers; PAH, pulmonary arterial hypertension.

To evaluate the impact of MPs on SSc pathogenesis, we evaluated if there were any associations and/or correlations between circulating MPs numbers and patients’ clinical parameters, particularly among patient not treated with methotrexate or tDMARDs. No correlation was observed between total circulatory MPs levels and mRSS, or the limited or diffuse form of the disease (median of 9815 MPs/µl *vs.* 9947 MPs/µl, respectively, data not shown), or even with the SSc serology (data not show). There were also no association between circulatory MPs levels and disease duration, presence of active smoking, heart disease, gastrointestinal involvement, synovitis or tenosynovitis (data not shown). We observed a weak but significant inverse correlation between Raynaud’s phenomenon duration and EMPs concentration (r=−0.369, p=0.01, data not shown) while no association were observed between SSc patients’ clinical parameters tested and PMPs levels (data not shown). Moreover, we observed a significant association between total MPs concentration and the pulmonary disease. Indeed, a higher concentration of MPs was observed for patients with ILD on HRCT, compared with other SSc patients and HD (median of 10880 MPs/µl vs 6700 MPs/µl vs 5331 MPs/µl, respectively, **Figure 2A**, p<0.05). Looking at patients without PAH, those with ILD present a higher EMPs concentration than the other SSc patients and HD (median of 69 EMP/µl vs 18 EMP/µl vs 37 EMP/µl, p<0.01 and p<0.05, respectively, Data not shown). Furthermore, SSc patients with lung fibrosis showed as well elevated MPs concentration compared to patients without lung fibrosis (median of 17,177 vs 8,052, respectively, **Figure 2B**, p<0.05). Accordingly, total MPs concentration was inversely correlated to SSc patients’ respiratory function tests (RFT). Particularly, total MPs levels were moderately but significantly inversely correlated with Total Lung Capacity (TLC; r=−0.3934, p<0,005, **Figure 2C**) and forced vital capacity (r=−0.4376, p<0,005, **Figure 2D**). Although weak, we also observed a significant inverse correlation of the total MPs concentration to the volume-corrected carbon monoxide diffusing capacity (DLCO/VA; r=−0.3274, **Figure 2E**, p<0.05). EMPs level was also weakly but significantly inversely correlated with FVC (r=−0.3312, p<0,05, data not shown). No other correlations between MPs, EMPs or PMPs with clinical features were observed. Vascular damage was also associated to total MPs levels, as they were significantly higher in SSc patients with active digital ulcers (DU), at the time of sampling, than in the rest of the patients (median of 27275 MPs/μl *vs*. 7504 MPs/μl, **Figure 2F**, p<0.01). Among patients without ILD, patients with high PAH were more likely to present high levels of MPs (median of 22237 MPs/μl *vs*. 6666 MPs/μl, p<0.05, respectively, **Figure 2G**) and high levels of EMPs (median of 152 EMPs/μl *vs*. 21 MPs/μl, p<0.01, respectively, Data not shown). Taken together, these results suggest that total circulating MPs and EMPs are associated with ILD and vascular damage.

### MPs From SSc Patients Induce a Profibrotic Profile in Human Dermal Fibroblasts

MPs were differently represented in HD and SSc patients and the accumulation of MPs in SSc patients correlates with certain clinical features associated with tissue fibrosis. Therefore, we assessed whether MPs may directly contribute to tissue fibrosis through the production of extra cellular matrix (ECM) by dermal fibroblasts. We thus examined the expression of the genes involved in collagen synthesis (Collagen Type I Alpha 1, Col1a1, and Collagen Type I Alpha 2, Col1a2) or its degradation (Matrix Metallopeptidase 1, MMP1) and in recruitment of immune cells (C-C Motif Chemokine Ligand 2, CCL2) in HD dermal fibroblasts upon stimulation with HD or SSc patients-derived MPs.

Dermal HD fibroblasts were first stimulated with the transforming growth factor (TGF)-β as a positive control. As expected, TGFβ induced Col1a1 (mean ± SEM of fold change: 2.33 ± 0.39-fold increase *vs*. the medium condition, **Figure S3A**) and Col1a2 (2.03 ± 0.34-fold increase *vs*. the medium condition, **Figure S3A**) mRNA expression in HD dermal fibroblasts while MMP1 and CCL2 were only slightly affected. To further address the reproducibility of the fibroblast co-culture with MPs, we repeated the co-culture three times, using fibroblasts from three healthy donors and different concentration of purified total MPs from a single healthy donor. We did not observe any significant variation in the expression of mRNAs coding for Col1a1, Col1a2 and CCL2 (**Figures S3B, C, E**). Concerning the expression of MMP1, we observed an increase of mRNA expression although not significant (mean ± sem of fold change for 10 MPs/µl: 1.62 ± 0.25; for 100 MPs/µl: 2.12 ± 0.34; for 1,000 MPs/µl: 1.50 ± 0.09, **Figure S2D**). These optimization steps, showed that fibroblasts responded as expected to TGFβ stimulation and that the impact of HD MPs on fibrotic properties was stable in our experimental settings, allowing us to further evaluate the fibrotic potential of SSc patients-derived MPs.

**Figure 3:**
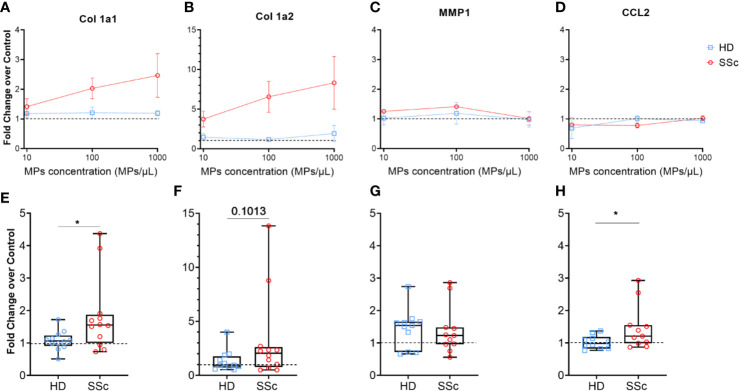
MPs activated human dermal fibroblasts to produce extracellular matrix. Levels of Col1A1 mRNA **(A, E)**, Col1A2 **(B, F)**, MMP1 **(C, G)** and CCL2 **(D, H)** expression were assessed in fibroblasts stimulated with an increasing concentration of MPs from HD (n = 3) and from SSc patients (n= 3) **(A–D)**, or with 1,000 MPs/µl of HD (n=12) or SSc (n=12) **(E–H)** by reverse transcriptase quantitative polymerase chain reaction (RT qPCR) and expressed in ΔΔCt toward housekeeping gene and DMEM 10% FBS. The dashed line represents the fibroblast condition treated only with DMEM 10% FBS; In **(A–D)**, Dot and error bars show the meanSEM. In **(E–H)**, Box-plots represent the extreme values, the first and third quartiles and the medians. *p < 0.05 **(A, D, E, H)** by Mann-Whitney test. MPs, microparticles; HD, healthy donors; SSc, systemic sclerosis; Col1a1, Type 1 Collagen a1; Col1a2, Type 1 Collagen a2; MMP1, Matrix Metallopeptidase 1; CCL2, C-C Motif Chemokine Ligand 2; FBS, fetal bovine serum.

Therefore, we stimulated HD dermal fibroblasts with increasing numbers of MPs from healthy donors and SSc patients (**Figure 3**). We tested MPs from three HD and three SSc patients at the three concentrations that were previously used during the optimization step. As expected, we did not observed any effect of MPs from HD on Col1a1 (mean ± sem of fold change for 10 MPs/µl: 1.18 ± 0.14; for 100 MPs/µl: 1.21 ± 0.19; for 1,000 MPs/µl: 1.19 ± 0.08, **Figure 3A**) and Col1a2 (mean ± sem of fold change for 10 MPs/µl: 1.46 ± 0.43; for 100 MPs/µl: 1.16 ± 0.16; for 1,000 MPs/µl: 1.81 ± 1.37, **Figure 3B**), at the tested concentrations. This was also true for MMP1 (mean ± sem of fold change for 10 MPs/µl: 1.02 ± 0.22; for 100 MPs/µl: 1.19 ± 0.36; for 1,000 MPs/µl: 0.97 ± 0.26, **Figure 3C**) and CCL2 expression (mean ± sem of fold change for 10 MPs/µl: 0.69 ± 0.35; for 100 MPs/µl: 1.01 ± 0.10; for 1,000 MPs/µl: 0.93 ± 0.06, **Figure 3D**).

Conversely, MPs from SSc patients upregulated Col1a1 (mean ± sem of fold change for 10 MPs/µl: 1.41 ± 0.37; for 100 MPs/µl: 2.03 ± 0.35; for 1,000 MPs/µl: 2.47 ± 0.7, **Figure 3A**) and Col1a2 expression (mean ± sem of fold change for 10 MPs/µl: 3.74 ± 1.00; for 100 MPs/µl: 6.56 ± 1.95; for 1,000 MPs/µl: 8.32 ± 3.32, **Figure 3B**) in a dose dependent manner. MMP1 (mean ± sem of fold change for 10 MPs/µl: 1.25 ± 0.05; for 100 MPs/µl: 1.41 ± 0.05; for 1,000 MPs/µl: 0.92 ± 0.19, **Figure 3C**) and CCL2 expression (mean ± sem of fold change for 10 MPs/µl: 0.80 ± 0.01; for 100 MPs/µl: 0.78 ± 0.08; for 1,000 MPs/µl: 1.23 ± 0.08, **Figure 3D**), as with HD MPs, were not affected by SSc MPs.

Given the dose dependent impact of SSc MPs on the fibrotic potential of fibroblasts, we decided to extend our analysis using the highest concentration of MPs (1,000/µl) to 12 HD and 12 SSc patients. We observed a significant increase of Col1a1 and CCL2 expression induced by MPs from SSc patients compared to HD (median of 1.55 *vs.* 1.06 for Col1a1, respectively, p<0.05, **Figure 3E**, and median of 1.21 *vs.* 0.98, for CCL2, respectively, p<0.05, **Figure 3H**), and a trend toward for the increased of Col1a2 expression induced by MPs from SSc patients compared to HD (median of 2.05 *vs.* 0.935, respectively, p=0.1, **Figure 3F**). We haven’t observed any difference between MPs from SSc patients and HD on MMP1 expression (median of 1.23 *vs.* 1.54, respectively, **Figure 3G**). Our findings thus suggest that MPs from SSc patients promote a pro-fibrotic response in human dermal fibroblasts.

### Differential Profibrotic Effects of MPs According to SSc Patients Disease Duration, Presence of Pulmonary Fibrosis, and Treatment With Methotrexate

We next investigated whether the profibrotic effect of MPs that we observed was associated to any clinical parameters in the 12 SSc patients tested. For this purpose, the profibrotic potential of MPs from HD and SSc patients was expressed as the ratios between Col1a1/MMP1 or Col1a2/MMP1 expression.

**Figure 4:**
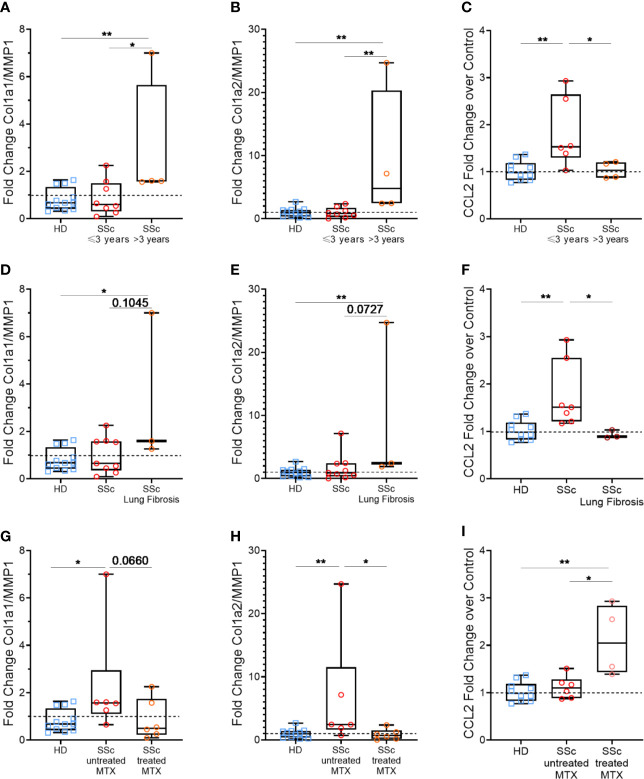
Effects of patients’ according to clinical features: disease duration, pulmonary fibrosis and methotrexate intake. Levels of Col1A1 mRNA, Col1A2, MMP1, and CCL2 were assessed in fibroblasts stimulated with 1,000 MPs/µl of HD (n=12) or SSc (n=12) by reverse transcriptase quantitative polymerase chain reaction (RT qPCR) and expressed as a Col1a1/MMP1 **(A, D, G)** or Col1a2/MMP1 ratio **(B, E, H)**, or in relation to ΔΔCt for housekeeping gene and for DMEM 10% FBS as the baseline condition for CCL2 **(C, F, I)**. Then, for each analysis, we compared the effect of MPs from HD or SSc patient according to different clinical parameters: duration between the onset of the first symptoms other than Raynaud’s phenomenon less than (n=8) or greater than (n=4) 3 years **(A–C)**, presence (n=3) or absence (n=9) of lung fibrosis **(D–F)** and patients treated (n=6) or not treated (n=6) with methotrexate **(G–I)**. The dashed line represents the fibroblast condition treated only with DMEM 10% FBS; Box-plots represent the extreme values, the first and third quartiles and the medians. **p < 0,01 **(A–C, E, F, H, I)**, *p < 0.05 **(A, C, D, F–I)** by Mann-Whitney test. MPs, microparticles; HD, healthy donors; SSc, systemic sclerosis; Col1a1, Type 1 Collagen a1; Col1a2, Type 1 Collagen a2; MMP1, Matrix Metallopeptidase 1; CCL2, C-C Motif Chemokine Ligand 2; MTX, methotrexate; FBS, fetal bovine serum.

First, we compared the MPs profibrotic effect based on whether they were coming from patients that have been diagnosed with another symptoms than Raynaud phenomenon for less than 3 years (early disease) or for more than 3 years (late disease). We observed that Col1a1/MMP1 and Col1a2/MMP1 ratios were increased when HD fibroblast were stimulated with MPs from SSc patients with a longer disease duration compared to those with a shorter disease duration (median of 1.60 *vs*. 0.60, respectively, p<0.05, **Figure 4A** and 4.80 *vs*. 0.82, p<0.01, respectively, **Figure 4B**). Conversely, we observed a statistically higher expression of CCL2 for patients with a shorter disease duration compared to patients with a longer disease duration, which showed a similar profile than healthy donors (median of 1.53 *vs*. 1.03 *vs*. 0.96, respectively, p<0.05 & p<0.005, **Figure 4C**).

Next, we distinguished between patients with and without pulmonary fibrosis. Similarly, MPs isolated from SSc patients with lung fibrosis showed a greater profibrotic profile, with an upward trend for Col1a1/MMP1 (median of 1.59 *vs*. 0.65, p=0.1, **Figure 4D**) and Col1a2/MMP1 ratio (median of 2.42 *vs*. 0.91, p=0.07, **Figure 4E**), but reduced ability to induce CCL2 (median of 0.89 *vs*. 1.51, p<0.05, **Figure 4F**).

Among the tested patients, none were treated by tDMARDs, but 6 was treated with methotrexate. Interestingly, when we compared MPs from patients treated or not with methotrexate, we observe the opposite results to our previous observations. Indeed, MPs from SSc patients treated with methotrexate showed a reduced profibrotic potential, with Col1a1/MMP1 and Col1a2/MMP1 ratios close to those of HD (median of 0.49 *vs*. 0.68, respectively, **Figure 4G** and median of 0.72 *vs.* 0.98, respectively, **Figure 4H**), while they upregulated CCL2 expression, compared to the untreated patients and HD (median of 2.05 vs. 1.10 vs. 0.96, respectively, p<0.05 and p<0.005, **Figure 4I**).

Thus, patients with a longer disease duration and with a lung fibrosis have MPs that display an elevated profibrotic potential, and treatment with methotrexate seems to limit MPs profibrotic potential.

## Discussion

In our study, we have shown that there is an increase in circulatory MPs in SSc patients especially those shedding from platelets, but not from EC, compared to HD. This increase is especially observed in patients without methotrexate or tDMARDs. We also demonstrated a profibrotic effect of MPs on dermal fibroblasts specifically of those from SSc patients.

Our results on PMPs were comparable to the study of Nomura et al. that showed increased levels of PMPs in 42 SSc patients compared to 30 HD (15). They also showed an increase in monocytes-derived MPs, however due to the detection of low number of MPs derived from immune cells, we were unable to quantify monocytes-derive MPs in our cohort.

Interestingly, we found in patients treated with immunosuppressive agents such as methotrexate or tDMARDs, that there was a decrease in circulatory PMP concentration. Two tDMARDs used in SSc patients, tocilizumab or baricitinib, target respectively IL6 receptor and the Jak1-2 Stat3 signaling pathway that indirectly affects IL6 and type I interferon signaling. Furthermore, methotrexate was also previously reported to reduce the IL6 expression in rheumatoid arthritis patients (27–29). It was previously reported that IL6 circulatory levels, in patients with coronary heart disease, are correlated to PMP and EMP levels (30). Therefore, blocking this IL6 pathway either directly or indirectly may affect MPs shedding.

Altogether, observations in human studies on MPs are heterogenous due to low number of patients, specific disease subtypes and technical procedures, but our results provide robust data on a large number of patients that reinforce and clarify existing data (31). Elevated levels of total MPs and EMPs were associated with an interstitial lung disease phenotype and were inversely correlated with respiratory function test, suggesting their involvement in the pathophysiology of the disease. Additionally, we confirmed that total MPs and EMPs counts were associated to PAH as already described on a very small cohort of SSC patients (17). Total MP were also associated to digital ulcers, reinforcing their potential role in the vasculopathy. Prospective studies are ongoing to identify whether more than being a witness, MPs levels could help to predict the onset of severe complications of SSc notably vascular such as PAH or digital ulcers. In the majority of our analyses, it is the total MPs level that is significantly associated with SSc patients’ clinical parameters studied, and to lesser extent EMPs. We did not find any association between circulatory PMP levels and the vascular or fibrotic features of SSc patients. Among the CD235a^-^ MPs, EMP and PMP proportion represent less 10-20% of total MPs, which means that the cellular origin of almost 80-90% MPs has unfortunately not been identified. Identifying the remaining fraction of MP would be of great interest to further identify the source of circulatory MPs that are pathogenic in SSc patients. Another hypothesis would be that, rather than the number of individual MPs, their content would be of major importance. Accordingly PMPs associated HMGB1was recently shown to contribute to vasculopathy during SSc by Maugeri et al. (16). PMPs from SSc patients induced neutrophil activation and the extrusion of NETS in a HMGB1-dependent manner which consequently induced endothelial cell damage and subsequent fibrosis in mice. However, the *in vivo* experiments were conducted using injection of human MPs in a mouse strain lacking adaptive immunity that limits the full elucidation of the role of MPs during the course of SSc either on vascular damage and fibrosis and no specific association between HMGB1 content on PMPs with SSc patients clinical outcomes were established. Therefore our results suggest a more direct role of MPs in SSc pathophysiology, however it will be of great importance to analyze MPs content in our patients and seek any association with clinical parameters in order to shed light on mechanisms of MPs profibrotic properties.

A previous study has described that EMPs from idiopathic pulmonary fibrosis patients stimulated the migration of normal human lung fibroblast (32) and other reports suggest direct implication of MP in fibrosis genesis, through their content or their surface markers [DAMPs, metabolite implied in the ROS production or nuclear factor B pathway, plasmin expression etc.; (33)]. Another recent observation, provided by Wermuth et al., show the profibrotic effect of exosomes from SSc patients (34). Interestingly, the authors showed that SSc exosome had a profibrotic effect in a dose response dependent, especially on Col1a1 expression. Here, we provide new evidence that purified MPs from SSc patient exert a pro-fibrotic impact on human primary fibroblasts compared to MPs from HD, notably in the induction of type 1 collagen expression, but also in the recruitment of the immune system by increasing CCL2 expression. Our results, in addition to exosome’s profibrotic effect already published, reinforce the role of extracellular vesicles, like exosomes or MPs, on fibrosis induction during SSc. Our *in-vitro* data are in accordance with our observations that increased circulatory MPs numbers in SSc patients are associated to lung fibrosis, such as fibrotic lung disease in HRCT, DLCO/VA and dyspnea. So far MPs role in SSc lung fibrosis was described to be indirect, through neutrophils activation (16) and endothelial cells damage being followed by pulmonary fibrosis (17). In accordance with our results, it was recently been demonstrated that plasma MPs containing mitochondrial DNA were increase in SSc patient with ILD, and stimulated inflammatory responses that may contribute to lung fibrosis (18). Our data shed new light on the potential direct contribution of MPs to lung fibrosis by modulating fibroblast activation.

Finally, we have observed differential pro-fibrotic effects of MPs according to clinical manifestations of SSc patients. Among them, MPs from patients with a longer disease duration or pulmonary fibrosis presented a higher profibrotic profile and a lesser capacity to express CCL2, implied in the recruitment of immune cells. Interestingly the response induced by MPs from patients treated with methotrexate on HD fibroblast did not impact CM production by fibroblasts as MPs purified from HD. Patients treated with methotrexate show less MPs or PMPs and those MPs are also less pro-fibrotic. Direct action of methotrexate on fibroblast-like synovial cells has already been described in rheumatoid arthritis (35), showing the importance of its action on the extracellular matrix, and this drug is given as a first-line treatment for early cutaneous sclerosis (36). We observed that methotrexate treatment increases the potential of SSc patients MPs to induce CCL2 a chemokine involved in the recruitment of immune cells, which may seem contradictory in light of what has already been described (37). One interesting avenue to study would be the impact of methotrexate on the MPs content, particularly their load of DNA. Methotrexate is a dihydrofolate reductase inhibitor, enzyme involved in the reduction of folic acid to tetrahydro-folic acid and then to folinic acid, and inhibits amido-phosphoryl transferase which converts phosphoribosyl-pyrophosphate to phosphoribosylamine, implied in the formation of inosine monophosphate (IMP). These two mechanisms of action leading to a decrease in the formation of purine base and thymidine, inhibit the formation of DNA and RNA. Given That, methotrexate may impact the overall DNA and RNA content of MPs. This observation is in line with the work of Ryu et al. on the link between MPs containing mitochondrial DNA from SSc patients and ILD (18). Wermuth et al. showed that exosomes purified from SSc patients have altered profile of microRNA (miRNA) derived from several anti and pro-fibrotic genes (34). They observed an increase of six profibrotic miRNA and a decrease of ten antifibrotic miRNA in SSc exosomes compared to HD. It is tempting to speculate that methotrexate might modify RNA or miRNA content in SSc derived exosomes. Whether MPs have similar miRNA alterations and whether methotrexate could impact is still to be explored. Although these results are in line with our observation indicating that MPs levels are associated with lung fibrosis, it appears that given the small number of patients tested (n=12), possible bias may exist. The representativity of patients in the study is not ideal, we need to increase the patients’ number, notably to ascertain the impact of methotrexate on MPs fibrotic potential. In addition, pro-fibrotic effect of the MPs that we showed was obtained using skin fibroblasts. However, our results show mostly an association with lung disease. Thus it would be relevant to address the impact of MPs also on lung fibroblasts. Apart from this direct role, it is worthwhile to pursue the study of the indirect and complementary role of MPs. Polymorphisms in the gene encoding the secreting desoxyribonuclease DNASE1L3 have been observed in both SSc and SLE diseases, suggesting a new potential common pathway important for the loss of tolerance (38, 39). DNASE1L3 is specifically secreted by macrophages and dendritic cells, and with DNase1, is responsible for the entirety of DNase activity in the serum (40). Sisirak et al. recently showed that the loss of DNASE1L3 in mice causes a rapid antibody response to double stranded DNA and chromatin (41). Mechanistically, DNASE1L3 was shown to digest chromatin in apoptotic cell-derived MPs, and its absence and/or downregulation caused the accumulation of DNA within MPs that ultimately lead to the break of tolerance to self-DNA. Reduction of DNASE1L3 activity in SSc could be responsible for the accumulation of immunogenic MPs that may represent the initial process governing the occurrence of inflammatory process in human, and eventually play a role as pro-fibrotic factors.

In conclusion, we show that circulatory MPs were increased in SSc patients, especially those with ILD, lung fibrosis and vasculopathy. Moreover, it seems that immunosuppressive treatments present an impact on their concentration. In contrast to HD MPs, MPs from SSc patients induced the extracellular matrix production and the CCL2 gene expression. To establish the potential of MPs as a biomarker in vascular or fibrotic phenotype in SSc, analysis of longitudinal cohorts should be performed to uncover potential correlations with disease severity or prognosis. Moreover, in-depth subtyping of the pro-fibrotic MPs and unraveling mechanisms involved in their production could pave the road to new therapeutic avenues.

## Data Availability Statement

All datasets generated for this study are included in the article/**Supplementary Material**.

## Ethics Statement

The studies involving human participants were reviewed and approved by CPP, SUD-EST IV, 18/024. The patients/participants provided their written informed consent to participate in this study.

## Author Contributions

DL, VS, CC-B, and M-ET designed the experiments. DL, ELe, and PL performed the experiments. VS, CR, ELa, PD, PB, CC-B, and M-ET wrote the paper. All authors contributed to the article and approved the submitted version.

## Funding

This work was supported by the Fondation pour la recherche Médicale (FRM) and the Association des Sclérodermiques de France (ASF).

## Conflict of Interest

The authors declare that the research was conducted in the absence of any commercial or financial relationships that could be construed as a potential conflict of interest.

## Supplementary Material

Supplementary Figure 1 Correlation between age and MPs levels. Levels of total MPs **(A, D)**, EMPs **(B, E)** and PMPs **(C, F)** from HD **(A–C)** and SSc patients **(D–F)** were put in relation to the age of the subject at the time of sampling. Correlation was determined using Spearman’s test. MPs, microparticles; EMPs,: endothelial cells derived microparticles; PMPs, platelets derived microparticles; HD, healthy donors; SSc, systemic sclerosis.

Supplementary Figure 2 Effects of immunosuppressant agents on level of circulatory MPs. Circulatory MPs and PMPs levels were compared between HD, SSc patients without immunosuppressive therapy, with steroids, hydroxychloroquine, mycophenolate mofetil, methotrexate and tDMARDs. Box-plot represents the extreme values, the first and third quartile and the median. ***p<0,0005 **(D)**, **p<0,001 **(C)** by Mann-Whitney. MPs, microparticles; PMPs, platelets derived microparticles; HD, healthy donors; SSc, systemic sclerosis; w/o IS, without immunosuppressor; HCQ, hydroxychloroquine; MMF, mycophenolate mofetil; MTX, methotrexate; tDMARD,: targeted disease-modifying antirheumatic drugs.

Supplementary Figure 3 Positive and reproducibility controls of fibroblasts and MPs co-culture. Levels of Col1A1 mRNA, Col1A2, MMP1 & CCL2 were assessed in fibroblasts stimulated with TGF as a positive control (10 ng/ml, n=4; **A**). The same levels of Col1A1 mRNA **(B)**, Col1A2 **(C)**, MMP1**(D)** & CCL2 **(E)** were assessed in fibroblasts stimulated with an increasing concentration of MPs of the same HD three times to test the reproducibility of the experience. The analysis was carried out by reverse transcriptase quantitative polymerase chain reaction (RT qPCR) and expressed in relation to ΔΔCt for DMEM as the baseline condition. The dashed line represents the fibroblast condition treated only with DMEM 10% FBS. Dot and error bars show the meanSEM. MPs, microparticles; Col1a1, Type 1 Collagen a1; Col1a2, Type 1 Collagen a2; TGFβ, Tumor Growth Factor ; MMP1, Matrix Metallopeptidase 1; CCL2, C-C Motif Chemokine Ligand 2; HD, healthy donors.
