# Supplementary figures and images for: Elevated Circulatory Levels of Microparticles Are Associated to Lung Fibrosis and Vasculopathy During Systemic Sclerosis
Source: Front Immunol. 2020 Oct 23;11:532177. doi: 10.3389/fimmu.2020.532177 (PMC7645042; doi:10.3389/fimmu.2020.532177)

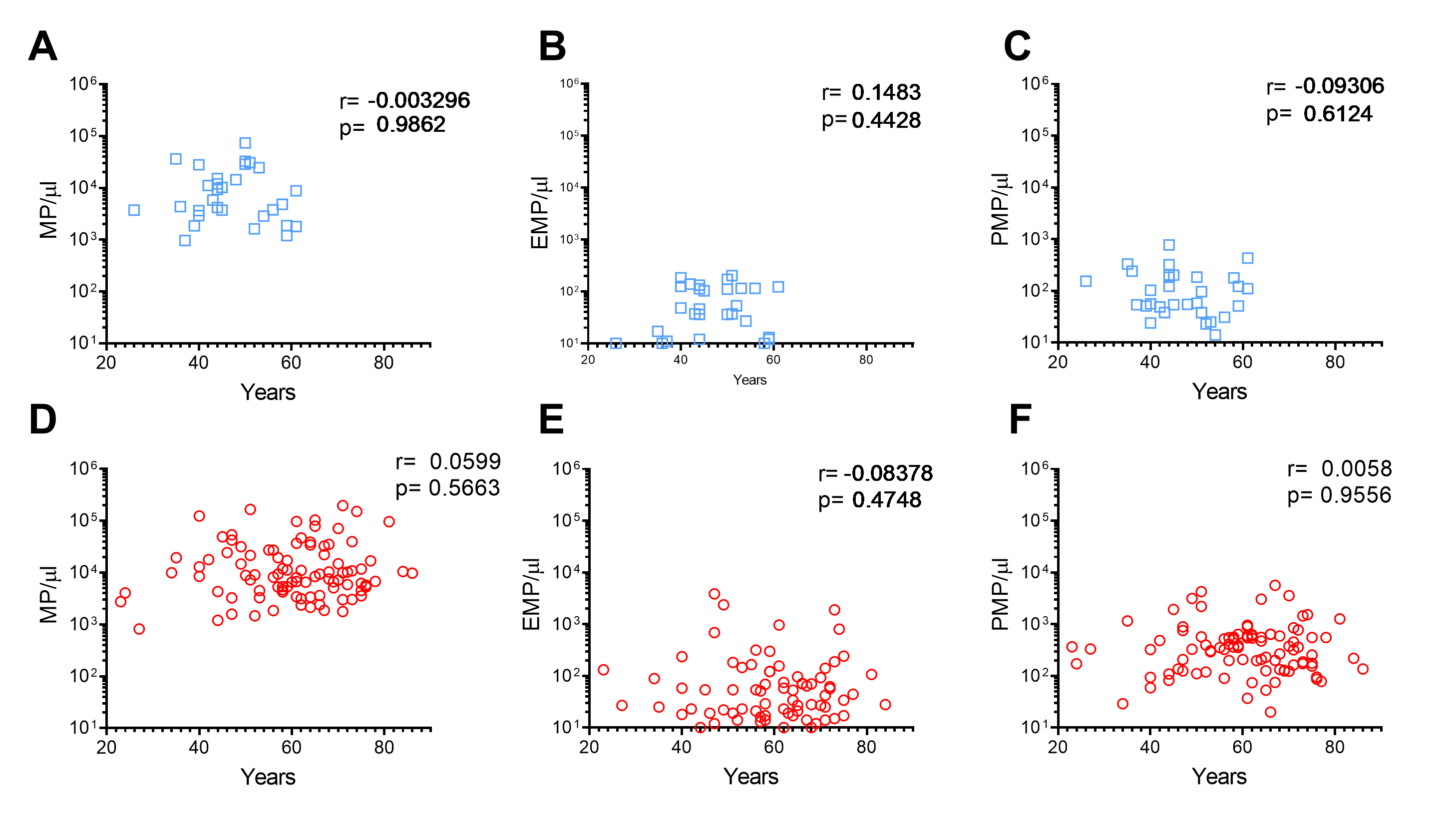

Supplement: Supplementary Figure 1 — Correlation between age and MPs levels. Levels of total MPs (A, D), EMPs (B, E) and PMPs (C, F) from HD (A–C) and SSc patients (D–F) were put in relation to the age of the subject at the time of sampling. Correlation was determined using Spearman’s test. MPs, microparticles; EMPs,: endothelial cells derived microparticles; PMPs, platelets derived microparticles; HD, healthy donors; SSc, systemic sclerosis. [file Image_1.tif]

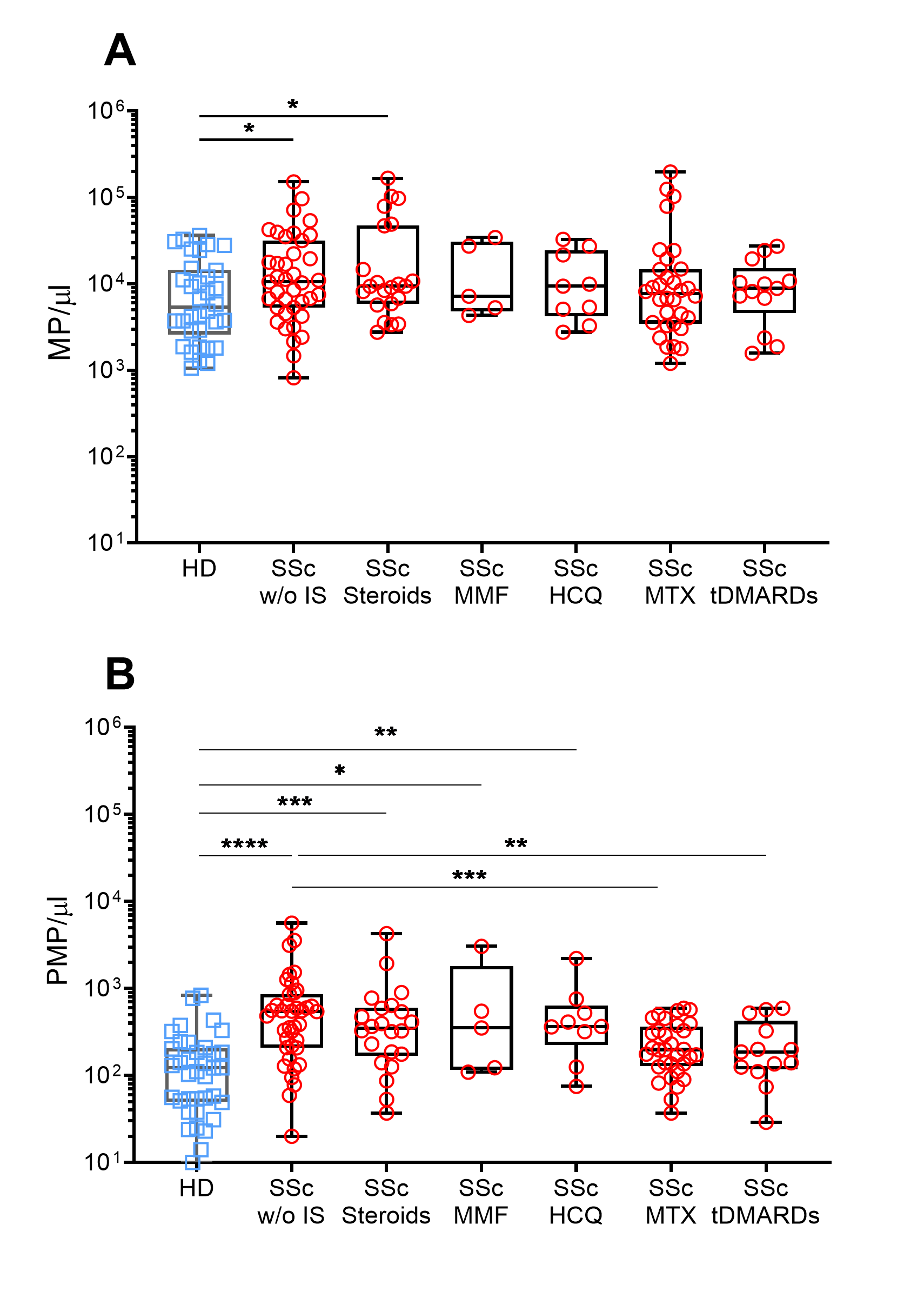

Supplement: Supplementary Figure 2 — Effects of immunosuppressant agents on level of circulatory MPs. Circulatory MPs and PMPs levels were compared between HD, SSc patients without immunosuppressive therapy, with steroids, hydroxychloroquine, mycophenolate mofetil, methotrexate and tDMARDs. Box-plot represents the extreme values, the first and third quartile and the median. ***p<0,0005 (D), **p<0,001 (C) by Mann-Whitney. MPs, microparticles; PMPs, platelets derived microparticles; HD, healthy donors; SSc, systemic sclerosis; w/o IS, without immunosuppressor; HCQ, hydroxychloroquine; MMF, mycophenolate mofetil; MTX, methotrexate; tDMARD,: targeted disease-modifying antirheumatic drugs. [file Image_2.tif]

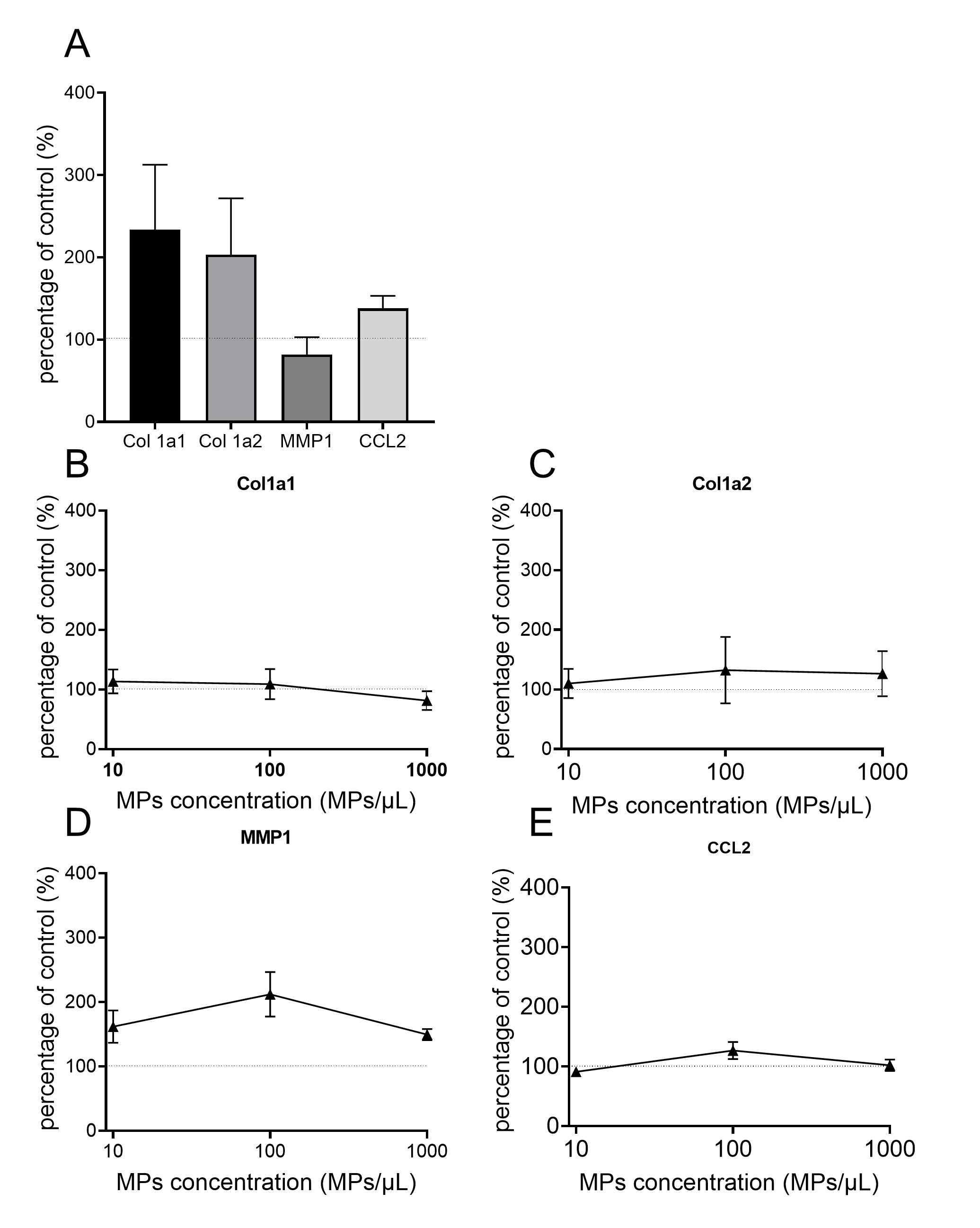

Supplement: Supplementary Figure 3 — Positive and reproducibility controls of fibroblasts and MPs co-culture. Levels of Col1A1 mRNA, Col1A2, MMP1 & CCL2 were assessed in fibroblasts stimulated with TGF as a positive control (10 ng/ml, n=4; A). The same levels of Col1A1 mRNA (B), Col1A2 (C), MMP1(D) & CCL2 (E) were assessed in fibroblasts stimulated with an increasing concentration of MPs of the same HD three times to test the reproducibility of the experience. The analysis was carried out by reverse transcriptase quantitative polymerase chain reaction (RT qPCR) and expressed in relation to ΔΔCt for DMEM as the baseline condition. The dashed line represents the fibroblast condition treated only with DMEM 10% FBS. Dot and error bars show the meanSEM. MPs, microparticles; Col1a1, Type 1 Collagen a1; Col1a2, Type 1 Collagen a2; TGFβ, Tumor Growth Factor ; MMP1, Matrix Metallopeptidase 1; CCL2, C-C Motif Chemokine Ligand 2; HD, healthy donors. [file Image_3.tif]
